# Value of CT Features in the Diagnosis of Papillary Thyroid Tumors in Incidental Thyroid Nodules

**DOI:** 10.1155/2020/9342317

**Published:** 2020-10-16

**Authors:** Fengyan Zhang, Ying Qiao, Hui Zhang

**Affiliations:** ^1^Department of Radiology, First Clinical Medical College, Shanxi Medical University, Taiyuan 030001, Shanxi Province, China; ^2^College of Medical Imaging, Shanxi Medical University, Taiyuan 030001, Shanxi Province, China

## Abstract

The present work has investigated the value of computed tomography (CT) features in the diagnosis of papillary thyroid tumors in the incidental thyroid nodules (ITNs). In the 82 enrolled patients, 101 thyroid nodules were incidentally found by the neck CT scanning, among which 49 were histologically confirmed to be papillary thyroid carcinoma (PTC) while the other 52 were nodular goiter (NG). The tumor location, size, shape, tiny calcification, cystic change, and signs of an irregular ring, marginal defects, and enhanced blurring were all depicted by CT features. Both univariate and multivariate analyses were employed to identify independent predictors of PTC. The univariate analysis of PTC and NG showed that four CT features were statistically significant, including tiny calcification and signs of an irregular ring, marginal defects, and enhanced blurring. Furthermore, the multivariate logistic regression model indicated that signs of an irregular ring, marginal defects, and enhanced blurring were strongly correlated with PTC, of which the odds ratio (OR) was 27.374 (95% CI: 5.871∼127.636), 28.587 (95% CI: 4.139∼197.460), and 4.315 (95% CI: 0.858∼21.694), respectively. In the predictive model of PTC, the value of sensitivity, specificity, accuracy, and Youden index was 87.8%, 94.2%, 91.1%, and 0.82 with a likelihood ratio of 15.1. Therefore, the signs of an irregular ring, marginal defects, and enhanced blurring may be helpful in the diagnosis of PTC in incidentally found thyroid nodules.

## 1. Introduction

Although sonography is the primary imaging test for a palpable thyroid nodule or known thyroid malignancy, thyroid abnormalities are oftentimes firstly detected by CT [1]. A thyroid nodule may be incidentally found by CT when it is used to evaluate an unknown bump at the neck area [2]. Sonography is known to suffer some limitations, such as the dependency on operators' skills and lack of advantages in determining the lymphatic metastasis in central groups [3, 4]. As a good supplement to sonography, CT can overcome the weakness of sonography [5–7]. Further understanding of CT in ITNs will be favorable to the early diagnosis and treatment of thyroid nodules [8, 9]. Papillary thyroid carcinoma (PTC) and nodular goiter (NG) are the most common malignant and benign thyroid nodules in ITNs [10–12]. Unfortunately, PTC and NG bear little characteristic difference in clinic imaging; they both display necrosis, cystic degeneration, calcification, and heterogeneous enhancement in imaging, which may lead to misdiagnosis in the clinic [13]. Previous works suggest that marginal defects and enhanced blurring were useful signs to the diagnosis of PTC [14]. However, the study upon the sign of an irregular ring is scanty, and the diagnostic value of multivariate analysis of multiple CT features in PTC has been rarely studied. Herein, we adopted a univariate analysis and a multivariate logistic regression model to explore the value of CT features in diagnosing PTC in incidentally found thyroid nodules.

## 2. Materials and Methods

### 2.1. Patients

**Figure 1:**
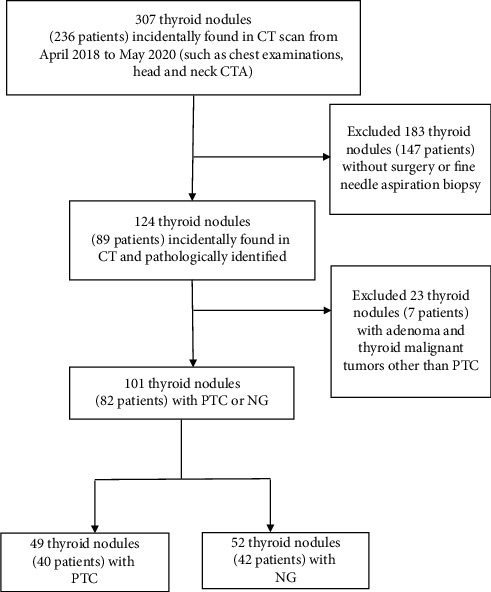
The patient selection and procedural flowchart.

Our institutional review board approved this study and waived the requirement for informed consent. The total number of enrolled patients is 82, consisting of 21 males (25.61%) and 61 females (74.39%). From April 2017 to May 2020, among the 124 incidentally found thyroid nodules in 82 patients, 101 thyroid nodules were subject to plain and enhanced CT scanning and pathologically confirmed as PTC or NG: 49 nodules were PTC and 52 nodules were NG (Figure 1). The clinical characteristics are shown in Table 1.

Inclusion criterion is thyroid nodules were accidentally found by plain and enhanced CT scanning. Exclusion criterion is pathologically confirmed thyroid nodules other than PTC and NG.

### 2.2. Examination Method

CT scanning was performed using SOMATOM Definition Force (Simens Health care, Forchheim, Germany). All patients were in a supine position for neck scanning. The patients were instructed to avoid swallowing during the scanning procedure [15]. The scanning parameters were set as following: automatic tube current adjust technology with 90 and Sn150 kV of A and B X-ray tube voltage; 0.25 s of frame rotation; 192 × 0.6 mm of collimation; 0.5 of dual-energy fusion coefficient; 2 mm of slice thickness; 0.75 mm of reconstruction increment. 60 ml of contrast medium, iodixanol (Hengrui Medicine, China), was bolus injected via a cubital vein by a power injector with a contrast medium concentration of 320 mg/ml at a rate of 3 mL/s. All nodules were confirmed by surgery or fine-needle aspiration within one month after the CT examination. The average of radiation dosage is 238.72 ± 6.53 mG*γ* cm of the dose-length-product and 1.03 ± 0.04 msv of the effective dose ED value.

### 2.3. Image Assessment

Two senior head and neck radiologists with more than 10 years of work experience have independently reviewed all results of CT scanning. Reviewers were blinded to pathological findings, while being informed that they were involved in a study upon the CT features of incidentally found thyroid nodules. Inter-reviewer disagreements were resolved by a third reviewer with 15 years of experience in head and neck radiology.

**Figure 2:**
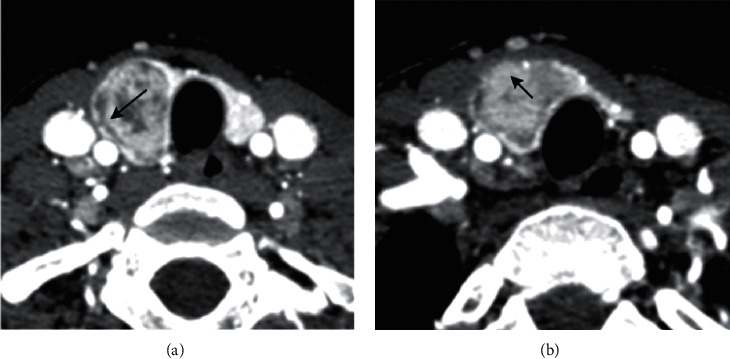
Low-density nodule in the right lobe of the thyroid, in a female, 27 years old. (a) The low-density ring sign has sharp horn protuberance at the edge (↑). (b) The lower level of (a) showing the irregularity of the low-density ring and the local interruption (↑).

**Figure 3:**
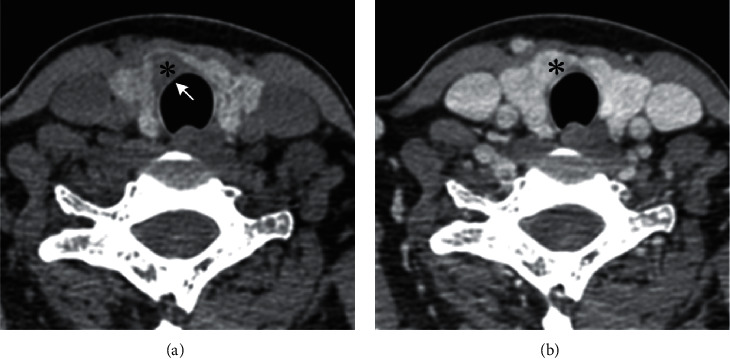
Right thyroid lobe near isthmus showing a low-density nodule (^*∗*^), in a female, 39 years old. (a) Thyroid marginal interruption with “marginal defect sign” (↑). (b) The enhancement in blur sign and the decrease in the density difference between the lesion and the normal thyroid gland. The pathological diagnosis is PTC.

CT features including location (right/left/isthmus), size (maximum long-axis diameter), shape (regular/ irregular), tiny calcification (diameter less than 2 mm), cystic change, the sign of rings (an irregular/regular), the sign of margins (defect/continuous), and the sign of enhancement (blurring/clear) were measured and analyzed for all patients. The sign of an irregular ring refers to a low-density ring on the edge of thyroid nodules with an irregular margin (Figure 2), while the sign of a regular ring has a smooth margin. The sign of marginal defects was defined as a nodule located at the edge of or partly outside the thyroid. As the density of nodules is lower than that of the thyroid, it looks like a high-density thyroid defect (Figure 3(a)). The sign of continuous margin means that the thyroid margin is continuous. The sign of enhanced blurring is determined if a clear thyroid nodule on plain scanning becomes fuzzy after enhancement. This is because the difference in density between nodules and adjacent thyroid tissue from contrast-enhanced CT scanning was smaller than that from plain CT scanning (Figures 3(a) and 3(b)). On the other hand, the sign of enhanced clearness refers to that the nodule becomes clearer after enhancement.

### 2.4. Statistical Analysis

All statistical analyses were performed using SPSS (24.0. IL, USA). Continuous variables were presented as mean ± standard deviation. Differences in CT features between PTC and NG were compared using the chi-square test (categorical data) and Student's *t*-test (continuous variables). Multivariate logistic regression analyses were applied to identify independent predictors of PTC. Factors with *P* < 0.05 on univariate analysis were used as input variables for multiple logistic regression analysis, with the final model using Ward's advance method for selection. The probability value of the prediction model *P* > 0.05 was used as a cutoff value to calculate sensitivity (Se), specificity (Sp), accuracy, Youden index, and likelihood ratio. Efficiency refers to the percentage of the total number of cases with the correct diagnosis. Youden index = Se + Sp − 1; likelihood ratio = Se/1 − Sp.

## 3. Results

### 3.1. Univariate Analysis

Univariate analysis revealed that four CT features including tiny calcification and the signs of an irregular ring, marginal defects, and enhanced blurring differed significantly between PTC and NG. Notably, the sign of an irregular ring shows the highest sensitivity (87.8%) while the sign of marginal defects displays the best specificity as high as 96.2% (Table 2).

### 3.2. Multivariate Logistic Regression Analysis

**Figure 4:**
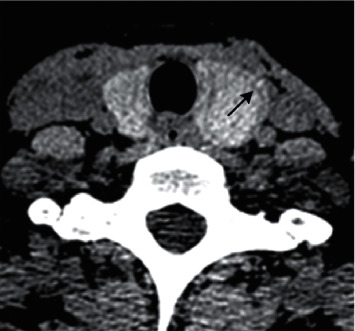
Thyroid left lobe nodule, in a female, 45 years old. Plain CT scan showing smooth low-density ring sign (↑) around the nodule, pathologically confirmed as nodular goiter.

**Figure 5:**
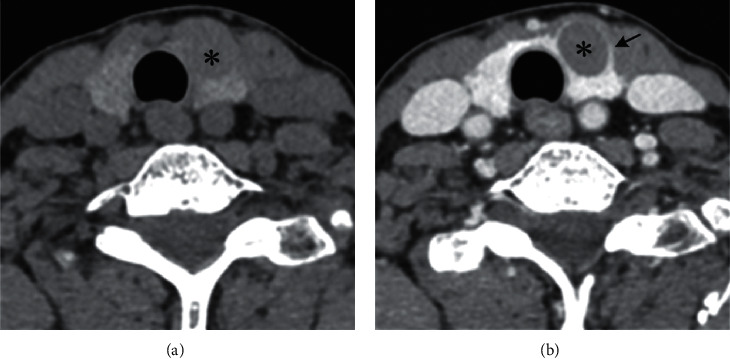
Low-density nodule (^*∗*^) in the left lobe of the thyroid gland, in a female, 72 years old. (a) Plain scan and (b) enhancement scan, which show that the lesion is clear after enhancement, and the difference between the density of the thyroid nodule and the normal thyroid is enlarged. At the same time, the edge of the thyroid gland was continuous (↑), pathologically confirmed as nodular goiter.

With the variety of thyroid nodules (PTC = 1, NG = 0) as a dependent variable and the four CT signs as independent variables: the sign of a ring was *X*1 (an irregular ring = 1 (Figure 2)), regular ring = 0 (Figure 4), the sign of the margin was *X*2 (defective = 1 (Figure 3(a)), continuous = 0 (Figure 5(b))), the sign of an enhanced blurring was *X*3 (blurring = 1 (Figures 3(a) and 3(b)), clearness = 0 (Figures 5(a) and 5(b))), and the calcification was *X*4 (tiny = 1, none tiny = 0). The logistic regression analysis was executed to establish the benign and malignant nodules predict mode with calcification excluded. Results indicate that the correlation between three signs and PTC was statistically significant. The thyroid nodules with signs of an irregular ring, marginal defects, and enhanced blurring have a high possibility of PTC with the risk increased 26.374 times, 27.587 times, and 3.315 times, respectively (Table 3). Based on the three signs derived from CT scanning, the specificity and the sensitivity of the logistic regression PTC prediction model reached 94.2% and 87%, respectively. The coincidence rate, Youden index, and likelihood ratio were 91.1%, 0.82, and 15.1, respectively, suggesting a high diagnostic accuracy of this model (Table 4).

## 4. Discussion

With the increase in incidental thyroid nodules, early diagnosis is of great significance to the selection of therapeutic regimens and the prognosis of tumors. In ultrasonic diagnosis, Cappelli et al. [16] reported that microcalcification is the most specific indicator for the diagnosis of PTC, with a specificity of 93% to 95%. This is consistent with our univariate analysis, which showed that the specificity of microcalcification was 94.2%. Notably, the microcalcification was excluded from the model in regression analysis. It may be because that CT is not as good as ultrasound in recognizing microcalcification.

The sign of marginal defects is defined as discontinuous margins at the periphery of normal high-density thyroids or defects in CT scanning caused by the abnormal size of nodules at the periphery or outside area of thyroids [14, 17]. The largest NG oftentimes is located in the interior area of a gland. The enhanced CT scanning can still recognize a small amount of normal tissue outside the lesion even if the lesion is located at the edge of a gland [18]. It may be because PTC tends to be more advanced when PTC invades neighboring organizations. According to the multivariate analysis of this group, the sign of marginal defects represents a risk factor for PTC, with an OR value of 28.587. This suggests that in the presence of thyroid nodules diagnosed with marginal defects, the probability of PTC tends to increase by 27.587 times. The sign of marginal defect also has a high specificity (96.2%) for the diagnosis of PTC. This agrees well with the report by Qu et al. [19] who had examined 524 thyroid nodules and found that the sign of marginal defects was highly specific to thyroid papillary carcinoma (92%). Hence, the sign of marginal defects plays an important role in the diagnosis of PTC.

The sign of enhanced blurring refers to the observation that the difference in the density between nodules and thyroid tissues of contrast enhancement is smaller than that of plain CT scanning [14]. Histologically, normal thyroid tissue is composed of hair follicles, the space between which is capillary beds. In contrast, PTC tissue is mainly composed of cells and fibers [20]. Due to the low iodine content of tumor cells, a significant difference in the density between tumor cells and normal tissues can be revealed by plain CT scanning. Besides, the axis of the PTC nipple consists of capillaries, generating a more phenomenal enhancement. The difference in density between tumors and normal thyroids is reduced, and nodules are relatively fuzzy compared to those observed in the plain CT scanning. Zhang et al. [21] showed that compared to enhanced ultrasound, most benign nodules displayed annular enhancement while most malignant nodules showed inhomogeneous enhancement. However, there is some overlap in the enhanced features of benign and malignant nodules.

The sign of a ring refers to the low-density ring located at the periphery area of a thyroid nodule. The low-density ring could be the capsule of lesions or the reactive fibrosis belt [22]. It was reported that PTCs have no real capsule. However, some fibers that are scattered in the fibrosis area may pass through the tumor from different directions or locate around the nodules, forming a fake capsule [23]. Additionally, the quantity of cells in the fibrosis area is small, resulting in the observation of the low-density ring in enhanced scanning. Due to the different rates of tumor growth in diverse directions, the low-density fiber ring could be destroyed by tumor growth, after which new fiber rings will form again surrounding the new tumor. As a result, the low-density ring of PTC usually bears an irregular margin while that of NG has a regular margin due to the complete reactive fiber ring at the periphery area of NG. Compared with the sign of enhanced blurring and marginal defects, the sign of a ring has improved sensitivity and specificity in distinguishing PTC and NG.

Multivariate analysis manifested that the sign of an irregular margin ring was a risk factor of PTC with an OR value of 27.374, which indicated that the possibility of PTC diagnosis increased 26.374 times when there is a nodule with an irregular margin ring sign. Furthermore, the combination of these three signs improved the accuracy of PTC diagnosis regarding specificity (94.2%), sensitivity (87.8%), accuracy (91.1%), Youden index (0.82), and likelihood ratio (15.1).

The diagnostic efficiency of US-FNAB for thyroid nodules reported by Zhou et al. [24] has a sensitivity of 91.7%, a specificity of 95.3%, and an accuracy of 94.3%. The accuracy of our diagnostic model is slightly lower than that of US-FNAB.

This study has several limitations: first, most patients enrolled in this study came from the same local places, which might lead to selection bias in the results; second, no further analysis of these different nodule sizes was performed due to the small number of ITNs; third, other types of benign and malignant nodules were not investigated.

In the current study, we adopted a dual-energy scanning mode. In the future work, we are planning to use some dual-energy parameters such as iodine value and dual-energy curve to support the diagnosis of thyroid papillary carcinoma and lymph node metastasis.

## 5. Conclusions

In summary, the detection and differentiation of thyroid nodules can be highly elusive in the absence of guidance from CT scanning. With increasing involvement of CT in the clinic, it is crucial to make a preliminary determination upon the value of CT features in assessing ITNs. The signs of an irregular margin ring, marginal defects, and enhanced blurring are expected to provide substantial help to the diagnosis of PTC, which is the most common malignant thyroid nodule.

## Figures and Tables

**Table 1:** Clinical characteristics of thyroid nodules.

Variable	PTC	NG	*P* value
No. of patients	40	42	
Median age (year)	42.12 (16–73)	56.98 (23–78)	0.680
Sex			0.702
Female	29 (72.5%)	32 (76.2%)	
Male	11 (27.5%)	10 (23.8%)	
No. of nodules	49	52	
Diagnostic technique			0.094
Fine-needle punctuate	27 (55.1%)	37 (71.2%)	
Surgery	22 (44.9%)	15 (28.8%)	

PTC: papillary thyroid carcinoma; NG: nodular goiter.

**Table 2:** Univariate analysis of the characteristics and imaging signs of thyroid nodules.

Characteristics	PTC	NG	*P* value	Kappa
Location			0.326	NA
Right	23 (46.9%)	24 (46.2%)		
Left	24 (49.0%)	28 (53.8%)		
Isthmus	2 (4.1%)			
Long-axis diameter (cm)	2.57 ± 0.98	3.01 ± 2.06	0.371	NA
Shape			0.06	0.803
Regular	21 (42.9%)	32 (61.5%)		
Irregular	28 (57.1%)	20 (38.5%)		
Tiny calcification			0.028	0.779
Yes	10 (20.4%)	3 (5.8%)		
No	39 (79.6%)	49 (94.2%)		
Margin			0.00	0.811
Continuous	12 (24.5%)	50 (96.2%)		
Defect	37 (75.5%)	2 (3.8%)		
Enhancement			0.00	0.898
Blur	12 (24.5%)	32 (61.5%)		
Clear	37 (75.5%)	20 (38.5%)		
Cystic change			0.793	0.737
Yes	27 (55.1%)	30 (57.7%)		
No	22 (44.9%)	22 (42.3%)		
Ring sign			0.00	0.854
Regular	6 (12.2%)	48 (92.3%)		
Irregular	43 (87.8%)	4 (7.7%)		

Data in parentheses are percentages; NA: not applicable; kappa value represents the consistency between the two observers.

**Table 3:** Multivariate regression analysis of three signs.

CT signs	$\hat{\beta }$	SE	Wald	*P*	OR	OR 95% CI	
						Lower limit	Upper limit
Irregular margin ring sign	3.310	0.786	17.752	<0.001	27.374	5.871	127.636
Margin defect sign	3.353	0.986	11.563	0.001	28.587	4.139	197.460
Enhancement blur sign	1.462	0.824	3.148	0.076	4.315	0.858	21.694

$\hat{\beta }$, regression coefficient; SE, standard error; OR, odds ratio; CI, confidence interval; Wald, Wald chi-square value.

**Table 4:** Diagnostic efficacy of various signs.

CT signs	Sensitivity (%)	Specificity (%)	Accuracy (%)	Youden index	Likelihood ratio
Irregular margin ring sign	87.8	92.3	90.0	0.80	11.4
Margin defects sign	75.5	96.2	86.1	0.72	19.9
Enhancement blur sign	80.5	61.5	68.3	0.37	1.96
Logistic regression model	87.8	94.2	91.1	0.82	15.1

## Data Availability

All data used to support the findings of this study are included within the article.

## References

[1] Hoang J. K., Branstetter B. F. I., Gafton A. R., Lee W. K., Glastonbury C. M. (2013). Imaging of thyroid carcinoma with CT and MRI: approaches to common scenarios. *Cancer Imaging*.

[2] Sinnott J. D., Mortimer R., Smith J. (2017). The effect of routine radiological reporting of thyroid incidentalomas on rates of thyroid needle biopsy, thyroid surgery and detection of thyroid malignancy. *Clinical Endocrinology*.

[3] Moon W.-J., Jung S. L., Lee J. H. (2008). Benign and malignant thyroid nodules: US differentiation-multicenter retrospective study. *Radiology*.

[4] Frates M. C., Benson C. B., Charboneau J. W. (2005). Management of thyroid nodules detected at US: society of radiologists in ultrasound consensus conference statement. *Radiology*.

[5] Yang T.-T., Huang Y., Jing X., Gai X.-J., Li W. (2016). CT-detected solitary thyroid calcification: an important imaging feature for papillary carcinoma. *OncoTargets and Therapy*.

[6] May M. S., Wiesmueller M., Heiss R. (2019). Comparison of dual- and single-source dual-energy CT in head and neck imaging. *European Radiology*.

[7] Hoang J. K., Nguyen X. V. (2017). Understanding the risks and harms of management of incidental thyroid nodules. *JAMA Otolaryngology-Head & Neck Surgery*.

[8] Peng W., Liu C., Xia S., Shao D., Chen Y., Liu R. (2017). Thyroid nodule recognition in computed tomography using first order statistics. *Biomedical Engineering Online*.

[9] Park J. Y., Lee K. H., Cho S. G., Kim Y. J., Lee H. Y., Hong I. K. (2017). Incidental thyroid nodules on thoracic contrast-enhanced computed tomography in clinical practice during a 10-year period: characteristics, clinical outcomes, and factors contributing to further evaluation. *Medicine (Baltimore)*.

[10] Zhang L.-X., Xiang J.-J., Wei P.-Y. (2018). Diagnostic value of computed tomography (CT) histogram analysis in thyroid benign solitary coarse calcification nodules. *Journal of Zhejiang University-Science B*.

[11] Jebreel A. e., England J., Bedford K., Murphy J., Karsai L., Atkin S. (2007). Vascular endothelial growth factor (VEGF), VEGF receptors expression and microvascular density in benign and malignant thyroid diseases. *International Journal of Experimental Pathology*.

[12] Lim H. K., Park S. T., Ha H., Choi S. Y. (2016). Thyroid nodules detected by contrast-enhanced magnetic resonance angiography: prevalence and clinical significance. *PLoS One*.

[13] Cappola A. R. (2017). How to look for thyroid cancer. *Journal of the American Medical Association*.

[14] Han Z. J., Chen W. H., Shu Y. Y., Lai X. F., Xiang J. J. (2013). Value of CT in the differential diagnosis of papillary thyroid microcarcinoma and micronodular goiters. *Journal of China Clinic Medical Imaging*.

[15] Forghani R., Srinivasan A., Forghani B. (2017). Advanced tissue characterization and texture analysis using dual-energy computed tomography. *Neuroimaging Clinics of North America*.

[16] Cappelli C., Castellano M., Pirola I. (2006). Thyroid nodule shape suggests malignancy. *European Journal of Endocrinology*.

[17] Tanpitukpongse T. P., Grady A. T., Sosa J. A., Eastwood J. D., Choudhury K. R., Hoang J. K. (2015). Incidental thyroid nodules on CT or MRI: discordance between what we report and what receives workup. *American Journal of Roentgenology*.

[18] Yeom J. A., Roh J., Jeong Y. J. (2019). Ultra-low-dose neck CT with low-dose contrast material for preoperative staging of thyroid cancer: image quality and diagnostic performance. *American Journal of Roentgenology*.

[19] Qu J., Zhu M., Han Z. (2015). Value of CT features in diagnosing papillary thyroid microcarcinoma. *Journal of Diagnostic Imaging & Interventional Radiology*.

[20] Luo D., Li L. (2017). The progress of thyroid disorders radiology diagnosis. *International Journal of Medical Radiology*.

[21] Zhang B., Jiang Y.-X., Liu J.-B. (2010). Utility of contrast-enhanced ultrasound for evaluation of thyroid nodules. *Thyroid*.

[22] Li M., Zheng X., Li J. (2012). Dual-energy computed tomography imaging of thyroid nodule specimens. *Investigative Radiology*.

[23] Tamhane S., Gharib H. (2016). Thyroid nodule update on diagnosis and management. *Clin Diabetes Endocrinol*.

[24] Zhou W., Ni X., Ye T. (2014). Sonographic evaluation and ultrasound-guided fine-needle aspiration biopsy of thyroid nodules smaller than 5 mm in the maximum diameter. *Chinese Journal of Ultrasound in Medicine*.

